# Association between stairs in the home and instrumental activities of daily living among community-dwelling older adults

**DOI:** 10.1186/s12877-018-0830-3

**Published:** 2018-06-04

**Authors:** Kimiko Tomioka, Norio Kurumatani, Hiroshi Hosoi

**Affiliations:** Nara Prefectural Health Research Center, Nara Medical University, Shijo-cho 840, Kashihara City, Nara, 634-8521 Japan

**Keywords:** Home type, Instrumental activities of daily living, Older people, Stairs, Successful aging

## Abstract

**Background:**

There is insufficient evidence regarding the relationship of home environment with functional capacity among community-dwelling older people without disabilities. We conducted a population-based longitudinal cohort study and examined whether stairs in the home were associated with capability to perform instrumental activities of daily living (IADL) in community-dwelling high-functioning older adults.

**Methods:**

The target population was individuals aged 65 years or older living in two municipalities in Nara Prefecture in Japan. At the baseline survey, residents who were independent in IADL (*n* = 6722) were included as survey subjects. Subjects were divided into three groups according to their home type; one-storey residences, walk-up residences, or residences with an elevator. IADL was evaluated using the Tokyo Metropolitan Institute of Gerontology Index of Competence. Multiple logistic regression analyses stratified by gender were used to calculate the odds ratio (OR) and a 95% confidence interval (CI) for a decline in IADL, with one-storey residences as a reference. Age, studied area, marital status, working status, self-perceived economic status, body mass index, chronic diseases, smoking, drinking, eating habits, basic activities of daily living, cognitive functioning, depression, self-rated health, and social participation were used as covariates.

**Results:**

During the 3-year follow-up, 11.6% of the subjects showed a decline in IADL. After adjusting for covariates, women who lived in walk-up residences had a lower risk for IADL decline (adjusted OR = 0.72, 95% CI = 0.52–0.99), while living in a home with an elevator was not associated with IADL decline (adjusted OR = 0.94, 95% CI = 0.49–1.77). In contrast, there was no association between home type and IADL decline in men (walk-up residences, adjusted OR = 0.90, 95% CI = 0.71–1.14; residences with an elevator, adjusted OR = 0.82, 95% CI = 0.39–1.72).

**Conclusions:**

The presence of stairs in the home was associated with prevention of IADL decline over a 3-year period in older women without disabilities. Although a barrier-free house is recommended for older people, our findings indicate that a home with stairs may maintain the capability to perform IADL among older adults without disabilities.

**Electronic supplementary material:**

The online version of this article (10.1186/s12877-018-0830-3) contains supplementary material, which is available to authorized users.

## Background

According to a recent report by the United Nations [1], the proportion of people aged 65 or older in the total world population (population aging rate) increased from 5.1% in 1950 to 8.3% in 2015, and it is expected to rise to 18.1% in the year 2060. This indicates that the aging of society will rapidly increase in the latter half of this century. The aging population rate in Japan was 27.3% as of October 1, 2016 [2], and it was the highest in the world.

One of the social issues arising from this progressive ageing of society is the increasing number of older people who need nursing care (i.e., older people in need of nursing). In Japan, the government introduced the public nursing care insurance program in 2000 on the grounds of increasing need for caregiving services and the limitations for family members to give nursing care to other family members due to the changes in family structure [3]. At the time the program started, the number of older people assessed as being in need of nursing care under the public nursing care insurance program was 1.8 million. Since then, it has constantly increased, reaching to 6.2 million in 2015 [4]. It is expected the ratio of old-old people who are 75 or older will increase in the future, which will lead to further increases in the need for nursing care [1, 2].

Once an older person starts to suffer from disability, the decision about whether to live at home or move into a nursing facility becomes important. The rapid increase in the aging population has led to a shortage of nursing facilities for older people. Therefore, Japan and some European and American countries with high population aging rates are promoting home care as an alternative to institutional care as a part of measures to lower social security costs [5, 6]. In view of this social background, it has become a pressing issue to ensure housing stability so that older people can live in a familiar place in their own way until the end of their life, even if they end up needing nursing care. Therefore, studies regarding older people’s housing environments are receiving attention.

Regarding the effects on health of the home environment in older age, especially the impact of the home environment on healthy aging, some researchers have proposed that one of the important indicators is independence in activities of daily living (ADL) [7, 8], and they have reported on the associations between home environment and ADL-related outcomes. For example, Gitlin et al. carried out a randomized controlled trial with a group of older adults aged 70 and over who were not receiving home care services but who had functional difficulties, and reported on a multi-pronged home intervention, which included environmental modifications, and improvements in performing everyday activities, in community-dwelling older adults [9]. Hoenig et al. conducted a study with community-dwelling wheelchair users, and found that more environmental barriers predicted more restriction in daily activities such as medical and non-medical visits [10]. Iwarsson’s study targeted to individuals aged 75–84 living in their own homes, and reported that environmental barriers in the homes were related to ADL dependence [7]. Oswald et al. carried out a survey of people aged 75 to 89 years living alone in their own homes, and demonstrated that very old people living in homes with better accessibility had more independence in daily activities as well as a greater feeling of well-being [11]. These studies’ findings have pointed out that minimizing barriers in the home environment is vital for the older population to maintain their autonomy.

In contrast, recent reports have suggested that, in relation to health effects associated with the home environment, the presence or absence of functional limitations, rather than that of objectively measured environmental barriers, is an important component. For example, Tomsone et al. investigated the relationship between objective and perceived aspects of housing and perceived health among ADL independent and ADL dependent groups of older adults, and reported that persons with ADL dependence tended to have more functional limitations, poorer self-rated health, and poorer perceived housing such as more negative views of their housing situation and less attachment to their home than those without ADL dependence [12]. Ekström et al. studied the differences in the home and health situation between a younger old cohort (i.e., aged 67–70 years) and a very old cohort (i.e., aged 79–89 years), and found that very old people were less likely to live in a dwelling with objectively assessed environmental barriers, but more likely to have functional limitations and report accessibility problems in their housing compared to younger old persons [13]. Kylén et al. investigated objective and perceived aspects of home and health among older adults aged 67–70 living in ordinary houses, and found that all houses had some environmental barriers, but the participants had no problems with accessibility due to good health and functional capacity [14].

According to the World report on disability [15], stairs in a building can become environmental barriers to persons with disabilities and cause activity limitations and participation restrictions. However, if the building incorporates elevators and takes sufficient account of environmental factors by setting barrier-free conditions, activity limitations and participation restrictions can be reduced, as can the limitations to everyday life and social life. Based on the research studies [7, 9–11] cited above, stairs in buildings are a major environmental barrier to individuals with disabilities, and lead to dependence in ADL. However, based on studies [12–14], for individuals without disabilities, stairs in a building are unlikely to be a barrier. Instead, stairs in buildings may provide a good opportunity for older adults without disabilities to maintain their physical activity. That is, it is probable that the presence of stairs can create a favorable environment and maintain their functional capacity, which would otherwise deteriorate with advancing age. However, to our knowledge, there has been no study examining whether certain aspects of the home environment are associated with decline or maintenance in functional capacity in community-dwelling older people without disabilities.

We have therefore selected community-dwelling older adults without disabilities at baseline, and conducted a prospective cohort study to test a hypothesis that high-functioning older adults who live in a walk-up residence are less likely to experience a decline in functional capacity.

## Methods

### Study population

**Fig. 1:**
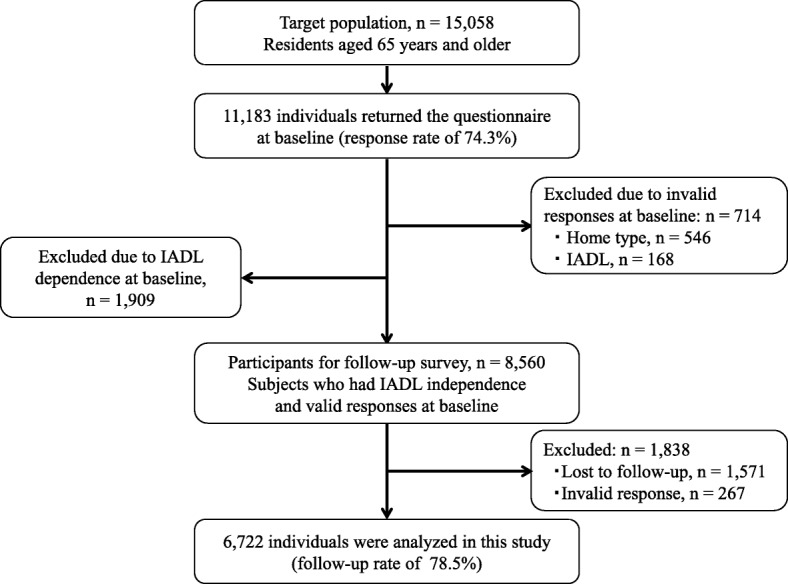
Flow chart of selection of analyzed and excluded subjects. IADL, instrumental activities of daily living

This study used data from a prospective cohort study targeting community-dwelling adults at least 65 years old [16–18]. The details of the target population were described in our previous study [18]. Briefly, a baseline survey was conducted in January to February of 2011 in two municipalities in Nara Prefecture, Japan. In one municipality (hereinafter A City), a medium-sized commuter town with an average Japanese population aging rate, 12,577 individuals who were not assessed as having “severe disabilities” by the public long-term care insurance system (i.e., care levels 3–5) were selected as survey subjects. In the other municipality (hereinafter B Town), a small-rural town with a declining birthrate problem and an aging population, all residents regardless of their care levels (*n* = 2481) were surveyed. Three years later, a follow-up study was conducted. Figure 1 displays a flow chart of the study population. In the baseline study, self-administered questionnaires were mailed to 15,058 residents, and returned by 11,183 persons (response rate of 74.3%). Of these, 546 subjects had invalid responses for home type and 168 subjects had invalid responses for IADL; they were excluded from the follow-up survey. Additionally, 1909 subjects who had already been classified as having IADL dependence were excluded from the follow-up. We followed the 8560 subjects with IADL independence for 3 years. After excluding 1838 individuals who could not provide valid follow-up data (1571 subjects who did not participate in the follow-up survey or 267 subjects who had invalid follow-up scores for IADL), 6722 subjects (2923 men and 3799 women) were analyzed for new-onset IADL decline; thus, among 8560 persons who had the follow-up survey, the follow-up rate was 78.5%.

Subjects who didn’t provide valid responses or were missing at follow-up (i.e., excluded subjects) tended to be male, were more likely to be older, have poorer BADL, poorer cognitive functioning, more depression, and poorer self-rated health than subjects who were included in the final analyses (i.e., analyzed subjects), (data shown in Table 1).

**Table 1:** Basic attributes of analyzed participants and subjects excluded due to invalid responses or lost to follow-up

	Analyzed subjects	Excluded subjects	*P* ^a^
	(*n* = 6722)	(*n* = 2552)	
Gender: men	43.5%	46.1%	0.023
Age: 75 years or older	36.6%	50.7%	< 0.001
Studied area: B Town	17.1%	18.8%	0.058
Subjects with poor BADL	19.1%	23.0%	< 0.001
Subjects with poor cognitive functioning	13.7%	20.5%	< 0.001
Subjects with depression	19.7%	27.5%	< 0.001
Subjects with poor self-rated health	12.7%	18.7%	< 0.001

BADL, basic activities of daily living

^a^Differences between the two groups were analyzed using Fisher’s exact test

### Assessment of functional capacity

The focus of this study was on instrumental activities of daily living (IADL), which is an index of complex daily living activities required of older adults living in the community [19]. IADL requires more complex and higher abilities than basic activities of daily living (BADL) such as toileting, feeding oneself, dressing oneself, walking, and bathing. Prior studies have reported that poor IADL can predict a decline in BADL and mortality [20, 21], and suggested that gender differences are a risk factor for IADL decline [16] and average years free of IADL disability [22].

In this study, IADL was assessed through the self-administered questionnaire, and measured using the Tokyo Metropolitan Institute of Gerontology Index of Competence (TMIG-IC) [23]. The TMIG-IC has an adequate validity and reliability for measuring the higher-level functional capacity of older adults [24]. The TMIG-IC is based on a subjective evaluation by the person him/herself, and has a multidimensional 13-item index with three subscales. The IADL subscale consists of five items: shopping, preparing meals, paying bills, banking, and using public transport. The response to each item is “yes” (able to do, 1 point) or “no” (unable to do, 0 points). The total score of the IADL subscale ranges from zero to five; the higher the score, the higher the IADL level. Subjects whose total score was 5 were classified as independence in IADL and those whose total score was ≤4 were classified as dependence in IADL [25]. We followed up the subjects who had IADL independence at the baseline for 3 years. Subjects who had IADL independence in the baseline study, but became IADL dependent by the follow-up study were defined as “subjects with IADL decline” [16, 17, 26]. This cutoff point was based on a prior study which reported that because the IADL subscale had high test–retest reliability, a variation of 1 point in IADL score was regarded not as a possible measurement error, but as a significant change in IADL [27].

### Assessment of home type

Type of housing was assessed from the self-administered questionnaire. Participants were asked, “Is your home (i.e., main living space) located on the second floor or higher?” Respondents were asked to answer either “Yes” or “No”. Following the first question, an additional question was asked to those who had replied that they were living on the second floor or higher; “Is there an elevator(s) installed at your residence?” Again, the respondents were asked to answer either “Yes” or “No”. These questions are commonly used to evaluate home type in studies targeting community-dwelling older adults in Japan [28]. On the basis of their responses, subjects were classified into three groups: those who lived in one-storey residences, those who lived in walk-up residences, and those who lived in residences with an elevator.

### Covariates

Through referencing prior studies [7–14, 29, 30], we selected the following variables for possible covariates that may correlate with home environment and IADL: socio-demographics (age, studied area, marital status, working status, and self-perceived economic status), health status (body mass index and self-reported physician-diagnosed chronic diseases), behavioral factors (drinking, smoking, and eating), a physiological factor (BADL), and psychological factors (cognitive functioning and depression). Because self-rated health [17, 29] and social participation [16] can predict a change in the IADL of community-dwelling older adults, these 2 variables (i.e., self-rated health and social participation) were added as covariates in the final model.

Working status at baseline was evaluated by asking a single question “Yes or no: do you currently have a paid job?” Responses were divided into working or not working. For self-perceived economic status, we asked respondents how they felt about their current financial state, giving them 4 answers to choose from: “very well set,” “somewhat well set,” “somewhat poor” and “poor”. Body mass index (BMI) was subdivided into normal (18.5 to < 25.0 kg/m^2^), underweight (< 18.5 kg/m^2^), and overweight (≥25.0 kg/m^2^) categories. For chronic diseases, subjects were asked if they were currently under medical treatment for the following diseases: hypertension, diabetes mellitus, cerebrovascular disease, and cancer. Responses were recorded as ‘present’ or ‘absent’. Alcohol consumption was divided into nondrinkers, social drinkers, occasional drinkers, or daily drinkers. Smoking history was categorized as never-smokers, ex-smokers, and current smokers. Assessment of eating habits was conducted using the Dietary Variety Score [31]; the total score ranges from 0 to 10, with higher scores expressing a higher level of dietary variety. The participants were divided into medians according to their scores; a high dietary variety group (a score of 7–10) and a low dietary variety group (a score of 6 or less). Assessment of BADL was conducted using the Barthel index [32]; the total score ranges from 0 to 100, with higher scores expressing a higher level of BADL. We defined a score of 100 as subjects with fully BADL dependence, and scores of < 100 as subjects with poor BADL. Assessment of cognitive functioning was conducted using the Cognitive Performance Scale [33]; the total score ranges from 0 to 6, with higher scores expressing a lower level of cognitive functioning. Because a prior study has demonstrated that a score ≥ 1 in the Cognitive Performance Scale can predict the onset of functional disability in community-dwelling older Japanese people [34], we defined a score of 0 as subjects with intact cognitive functioning, and scores of ≥1 as subjects with poor cognitive functioning. Assessment of depression was conducted using the 5-item short form of the Geriatric Depression Scale [35]; the total score ranges from 0 to 5, with higher scores defined as more depressed. We regarded scores of < 2 as subjects without depression, and scores of ≥2 as subjects with depression. Self-rated health was evaluated by asking a single question “How is your health in general? Is it very good, rather good, rather poor or very poor?” For social participation, we evaluated the total number of five types of social activities in which each respondent participated [16]: volunteer groups, hobby groups, senior citizens’ clubs, neighborhood community associations, and local events. We defined ≥1 activities as subjects with social participation, and zero activity as subjects without social participation.

All covariates except for BMI were dichotomized: age (65–74 vs. ≥75 years), studied area (A City vs. B Town), marital status (currently married or not), working status (currently working or not), self-perceived economic status (good (very/somewhat well set) vs. poor (very/somewhat poor)), BMI (normal, underweight, and overweight), chronic diseases (absent vs. present), alcohol intake (daily/occasional drinkers vs. non/social drinkers), smoking (ex/current smokers vs. never-smokers), eating habits (dietary variety high or low), BADL (fully independent vs. poor), cognitive functioning (intact vs. poor), depression (no depression vs. depression), self-rated health (good (very/rather good) vs. poor (very/rather poor)), and social participation (participation or not).

### Statistical analysis

We used multiple logistic regression analyses by the forced entry method, and calculated the odds ratio (OR) and a 95% confidence interval (CI) for a decline in IADL. An independent variable was the home type, with one-storey residences as the reference group. In Model 1, the adjusted ORs for age, studied area, marital status, working status, self-perceived economic status, BMI, chronic diseases, smoking, drinking, eating habits, BADL, cognitive functioning, and depression were estimated. In Model 2, self-rated health and social participation were added to the variables in Model 1. To test gender differences in the association between the home type and IADL decline, we performed stratified analysis by gender in both models.

In line with a statistical proposal on handling missing covariates [36], multiple imputations by chained equations were conducted. The independent variable, outcome, and all the covariate variables were entered into the imputation procedure. Within the study population, the covariates gender, age, and studies area had no missing values. Marital status (3.2% missing), working status (7.3% missing), self-perceived economic status (6.8% missing), BMI (1.8% missing), chronic diseases (8.9% missing), drinking (3.6% missing), smoking (4.7% missing), eating habits (5.1% missing), BADL (2.2% missing), cognitive functioning (2.6% missing), depression (3.3% missing), self-rated health (3.2% missing), and social participation (6.2% missing) were imputed as ordinal variables. We performed analyses on the complete pooled data set.

To evaluate the powerful influence of having stairs in the home on the prevention of IADL decline, population-attributable fraction (PAF) was calculated by using adjusted ORs based on the multiple logistic regression analyses. This method (i.e., the estimation of PAF based on OR) has been reported on by some researchers [37, 38]. In this study, we treated the PAF as the proportion of people for whom IADL decline would be expected to decrease if the entire population had stairs in the home, relative to the current pattern of home type.

We used IBM SPSS Statistics version 24.0 for statistical analyses with a significance level of 5%.

## Results

Among study participants (*n* = 6722), the average age at baseline was 73.1 (SD 5.8, range 65–96), with 43.5% being men. Regarding home type, 4849 (72.1%) were classified as subjects who lived in one-storey residences, 1705 (25.4%) as subjects who lived in walk-up residences, and 168 (2.5%) as subjects who lived in residences with an elevator. After a follow-up period of 3 years, 781 (11.6%) saw a decline in IADL.

Baseline characteristics of study participants according to home type are shown in Additional file 1. Subjects who lived in one-storey residences tended to be B Town residents. Subjects who lived in walk-up residences tended to be male, younger, married, working, drinkers, and smokers. Subjects who lived in residences with an elevator tended to have low dietary variety, depression, poor self-rated health, and no participation in social activities. The distribution of self-perceived economic status, BMI, chronic diseases, BADL, and cognitive functioning did not differ with respect to home type.

Baseline characteristics by the presence or absence of IADL decline according to gender are provided in Table 2. Regardless of gender, subjects who had IADL decline were significantly older, more likely to live in B Town, more likely to be underweight, less likely to be habitual drinkers, more likely to have poor BADL, poor cognitive functioning, depression, and poor self-rated health than those without IADL decline. However, the groups did not differ in the distribution of working status, diabetes mellitus, cancer, and smoking history. Men with IADL decline were less likely to have hypertension than men without IADL decline. Women with IADL decline were less likely to be married and poor economic status, were more likely to have cerebrovascular diseases, low dietary variety, and not participate in social activities compared to women without IADL decline.

**Table 2:** Baseline characteristics of study participants with and without IADL decline by gender (n = 6722)

	Men (*n* = 2923)			Women (*n* = 3799)		
	No decline (*n* = 2511)	Decline (*n* = 412)	*P* ^a^	No decline (*n* = 3430)	Decline (*n* = 369)	*P* ^a^
Age: 75 years and older	32.2%	49.0%	< 0.001	34.2%	75.6%	< 0.001
Studied area: B Town	14.5%	20.9%	0.001	17.8%	24.4%	0.003
Marital status: not married	12.0%	11.9%	1.000	35.1%	53.7%	< 0.001
Working status: not working	68.6%	65.8%	0.254	82.4%	81.3%	0.616
Self-perceived economy: poor	51.2%	53.4%	0.425	51.2%	45.3%	0.033
Body mass index						
Normal	75.4%	73.5%	< 0.001	73.6%	66.1%	< 0.001
Underweight	3.4%	7.8%		8.3%	14.6%	
Overweight	21.1%	18.7%		18.1%	19.2%	
Hypertension: present	40.2%	34.7%	0.039	39.5%	43.6%	0.131
Diabetes mellitus: present	14.1%	17.0%	0.130	8.7%	9.8%	0.499
Cerebrovascular diseases: present	3.3%	5.3%	0.063	1.8%	3.8%	0.016
Cancer: present	3.8%	3.9%	0.890	2.5%	1.6%	0.373
Alcohol: daily/occasional drinkers	63.4%	52.4%	< 0.001	18.8%	12.5%	0.002
Smoking: ex/current smokers	71.2%	69.2%	0.413	8.0%	7.0%	0.611
Eating: low dietary variety	49.5%	54.1%	0.089	34.1%	52.6%	< 0.001
Poor BADL	14.1%	21.6%	< 0.001	20.9%	35.0%	< 0.001
Poor cognitive functioning	12.4%	25.5%	< 0.001	11.8%	26.6%	< 0.001
Subjects with depression	16.5%	29.9%	< 0.001	18.8%	38.5%	< 0.001
Poor self-rated health	11.3%	19.2%	< 0.001	11.3%	28.5%	< 0.001
Social participation: none	30.8%	33.3%	0.330	27.9%	38.2%	< 0.001

BADL, basic activities of daily living; IADL, instrumental activities of daily living

^a^Differences between subjects with or without IADL decline were analyzed using Fisher’s exact test

Table 3 shows the ORs for IADL decline associated with home type according to gender. In Model 1, where the data were adjusted for socio-demographics, health status, and behavioral/physiological/psychological factors, among men, there was no association between home type and IADL decline, while women who lived in walk-up residences were less likely to have a decline in IADL. Significant association in women remained even after additional adjustment for self-rated health and social participation: in Model 2, among men, the ORs for IADL decline were 0.90 (95% CI: 0.71–1.14) in walk-up residences and 0.82 (95% CI: 0.39–1.72) in residences with an elevator. For women, the ORs for IADL decline were 0.72 (95% CI: 0.52–0.99) in walk-up residences and 0.94 (95% CI: 0.49–1.77) in residences with an elevator, compared to those who lived in one-storey residences. Additionally, to explore effect modification by gender, a logistic regression analysis including both “gender” and “the interaction term between gender and home type” was conducted. In Model 2, the results from this additional analysis were not significant (p for interaction = 0.258), suggesting that gender differences in the association between home type and IADL decline were unlikely. Regarding the contribution of the stairs to the decrease in the number of people with IADL decline, the PAF was 3.2% in men and 4.8% in women, suggesting that in this cohort, IADL decline might be preventable in 3.2% of males and 4.8% of females by having stairs in the home.

**Table 3:** Relationship between home type and IADL decline by gender

	Outcome^a^/Total^b^, n/n (%)		Adjusted OR (95% CI)	
			Model 1^c^	Model 2^d^
Men (n = 2923)				
One-storey residences	284/1918	(14.8)	1.00	1.00
Walk-up residences	119/941	(12.6)	0.90 (0.71–1.15)	0.90 (0.71–1.14)
Residences with an elevator	9/64	(14.1)	0.84 (0.40–1.76)	0.82 (0.39–1.72)
Women (n = 3799)				
One-storey residences	296/2931	(10.1)	1.00	1.00
Walk-up residences	59/764	(7.7)	0.72 (0.53–0.99)*	0.72 (0.52–0.99)*
Residences with an elevator	14/104	(13.5)	1.09 (0.58–2.04)	0.94 (0.49–1.77)

CI, confidence interval; IADL, instrumental activities of daily living; OR, odds ratio

^a^Number of people who developed IADL decline during the 3-year follow-up

^b^Number of people with IADL independence at baseline

^c^Adjusted for age, studied area, marital status, working status, self-perceived economic status, body mass index, chronic diseases, smoking, drinking, eating habits, basic activities of daily living, cognitive functioning, and depression

^d^In addition to Model 1, self-rated health and social participation were included

*p < 0.05

## Discussion

In this study, we investigated the association between home type and incident decline in IADL. We found that older women who resided in walk-up buildings were significantly less prone to have a 3-year decline in IADL compared to older women who lived in one-storey buildings. Although the mechanisms underlying the association between stairs in the home and IADL change are unknown, we have suggested the following. First, regarding the relation between housing environment and healthy aging, several theoretical approaches have been advocated [39–41]. In particular, much research has been based on the concept of Person-Environment Fit (PE-fit) [41]. The focus of PE-fit is on interactions and interdependencies between a person and his/her environment; the PE-fit idea assumes that optimal combinations of environmental conditions and person-based competencies can produce the best functioning for that person. As mentioned above, prior studies indicated that the objectively observed barriers in the home environment posed no problem for older adults without functional limitations [12–14]. Therefore, even if there are the stairs in their home, independent older adults may experience no negative effect on the practice of their daily activities, and have no disadvantage from environmental factors. Indeed, there is a possibility that houses with stairs have a more favorable PE-fit for older people without disabilities than barrier-free housing does. Second, physical inactivity has started to be recognized as the 4th biggest risk factor of human fatalities in the world, and regular physical activity is recommended not only for adults but also children and older people [42]. In so doing, people tend to focus on doing exercises, but it is also recommended to practice and review their daily/lifestyle activities. One guideline on physical activity has cited the following example; “taking the stairs instead of using the elevator, or walking to do errands instead of driving” [43]. Because older people spend more time in their homes compared to younger people [44], going up and down stairs at home may contribute a significant amount of the daily requirement for physical activity, resulting in a positive effect of housing with stairs on functional capacity. In particular, since women are traditionally the main housekeepers, they carry out daily activities like housekeeping, doing laundry, and going out for grocery shopping more than male members of the family [21, 45–47]. Such activities integrate physical activity into an older female’s daily life, and make a large contribution to their physical activity. Because regular physical activity has a beneficial effect on IADL disability for community-dwelling older persons [48], physical activity that comes from the use of stairs at home may help females maintain their IADL. On the other hand, men didn’t show significantly good effect on their IADL from going up and down stairs at home as women did, because men don’t do housekeeping tasks as often as women. Third, going up and down the stairs makes more demands on respiratory function, builds muscles in the lower leg, and engages greater complexity in neuropsychological organization than walking on flat surfaces and doing light exercises for older people. IADL is a higher-level functional capacity, and it doesn’t only require independent support of physical and cognitive functions but collaboration of each function [49]. The researchers consider that stimulation of muscles and nerve neurons encourages the multiple physical and mental functions required for IADL and leads to IADL maintenance, which is the mechanism matching the use-it-or-lose-it principle.

We need to consider the possibility of reverse causations and confounding factors in this study. First, community-dwelling older adults who were in the preclinical stage of IADL decline may be willing to live in buildings with an elevator. Although the relationship of housing type with BADL and cognitive functioning was not observed, subjects who lived in residences with an elevator were more likely to be older, suffer from depression, have poor self-rated health, and no participation in social activities (data is provided in Additional file 1). Therefore, we cannot dismiss the possibility that people who have a higher risk of developing IADL decline selectively live in buildings with an elevator. Second, because prior studies have demonstrated that regular exercise is positively associated with the maintenance of functioning in older adults [48, 50], we should have asked study participants a question about regular physical activity; our findings have the potential for confounding with fitness habits. Third, although the primary outcome (i.e., IADL) was assessed for both baseline and follow-up surveys, home type was evaluated only at baseline; it is possible that some of the participants’ residences may have been repaired or remodeled during our follow-up period. As home modifications can affect the autonomy of older people [8], it is necessary to conduct a prospective study focused on changes in home type during the follow-up period. Additionally, covariates were only assessed at baseline; we could not consider changing covariates during the follow-up period. Because chronic diseases, cognition, and marital status are identified as significant contributors to IADL in community-dwelling older adults [16, 51], it is possible that these factors impacted IADL if they changed during the follow-up period. However, this study did not take that into consideration. Because the results of this study cannot eliminate the possibility of reverse causation and confounding factors, it is necessary to be cautious when interpreting results.

Our study does not intend to emphasize the beneficial effect of stairs. The docility hypothesis states that people with lower capacity are more vulnerable to the demands of the environment than those with higher capacity [39]. Additionally, a literature search regarding the effects of home environments on disability-related outcomes has indicated that home environmental interventions, such as removing environmental barriers or installing supportive devices, are effective in improving daily activities among functionally vulnerable older adults [8]. Therefore, installing an elevator in a private home may prevent community-dwelling elderly with functional disabilities from being housebound, resulting in benefits to their everyday lives. We also accept that stairs and steps become environmental barriers to people with functional limitations, but in the cases of older people with higher levels of competence, we consider stairs in the home to lead them unconsciously to be more physically active and thereby their functional capacity.

Our study has some limitations. First, the assessment of home type was limited to questions concerning floor level and elevator installation. Previous studies have conducted a detailed assessment of the barriers in homes and accessibility [11, 13, 14]. Furthermore, it has been pointed out that both subjective and objective assessments of the home environment are important [7, 11–14], but there is no objective data collected for this study. Therefore, our assessment of home type is far from adequate. Further prospective cohort studies are required to confirm our findings by using more detailed and objective instruments to assess home environment. Second, in this study, subjects were asked whether they lived on the 2nd floor or higher in a building and those who answered they did not live higher than the 2nd floor were defined as residents living in one-floor housing. However, there is a possibility that some of those who are defined as living in one-floor housing may live in houses with basements including stairs. Also, there may be one-floor houses with stairs. The researchers cannot deny the possibility that the cohort living in a one-floor house in this study may include people living in a building with stairs. It is unclear whether this issue led to either an overestimate or underestimate of the association between home type and IADL. Third, although this study had a large sample sizes, the majority of older people lived in one-storey residences and the number of people who lived in residences with an elevator was very small. This can cause unstable results, with a wide range of 95% CIs. Additionally, although our study observed a significant association in women between stairs at home and IADL maintenance, its PAF was low (4.8%). The PAF estimate is determined according to the prevalence of cases (in this study, individuals with IADL decline) and the number of people with exposure (in this study, individuals living in houses with stairs). Because women were less likely to have IADL decline and live in walk-up residences than men, this may reduce the impact of stairs at home on IADL in women, leading to a low value of PAF. In the future, our findings need to be replicated in a prospective study in an area where a relatively large number of people live in residences with an elevator or in homes with stairs, for example, a major conurbation. Finally, this study could not yield a high rate of response and follow-up. It is possible that some of those who didn’t participate in the study simply couldn’t because their physical and mental functioning was poor. Regarding subjects who were excluded from this study, their physical and mental functioning were poorer than that of the analyzed subjects (data shown in Table 1). Because cognitive functioning [51] and self-rated health [17] are significant predictors of IADL decline, excluded subjects are considered to be a group at high risk of IADL decline. Additionally, subjects we could not follow-up on may include those who had moved to other dwellings or nursing homes due to a bad housing environment. Hence, subjects who had received a more negative impact of home type on IADL performance may have been selectively eliminated from the survey subjects in this study. These biases may lead to underestimating the association between home type and IADL.

## Conclusions

Despite the above-described limitations, to our knowledge, this is the first report to identify an association between home type and IADL of community-dwelling older people with a large-scale prospective cohort study. Although a barrier-free house is recommended for older people, our findings suggest that living in a home with stairs may have the benefit of IADL maintenance for older adults who are functionally independent.

## Additional file

Additional file 1: **Table S1.** Baseline characteristics of study participants according to home type (*n* = 6722). (DOCX 15 kb)

## Abbreviations

ADL: Activities of daily living
BADL: Basic activities of daily living
BMI: Body mass index
CI: Confidence interval
IADL: Instrumental activities of daily living
OR: Odds ratio
PAF: Population attributable fraction
PE-fit: Person-Environment Fit
TMIG-IC: Tokyo Metropolitan Institute of Gerontology Index of Competence
