# Supplementary material for: Association between stairs in the home and instrumental activities of daily living among community-dwelling older adults
Source: BMC Geriatr. 2018 Jun 4;18:132. doi: 10.1186/s12877-018-0830-3 (PMC6001070; doi:10.1186/s12877-018-0830-3)
Supplement: Supplementary file 1 — Table S1. Baseline characteristics of study participants according to home type (n = 6722). (DOCX 15 kb) [file 12877_2018_830_MOESM1_ESM.docx]

**Additional file 1: Table S1.** Baseline characteristics of study participants according to home type (n = 6722)

| Baseline characteristic | One-storey residences (n = 4849) | Walk-up residences (n = 1705) | Residences with an  elevator (n = 168) | *P^a^* |
| --- | --- | --- | --- | --- |
| Gender: men | 39.6% | 55.2% | 38.1% | <0.001 |
| Age: 75 years and older | 39.0% | 29.3% | 42.9% | <0.001 |
| Studied area: B Town | 18.3% | 14.1% | 14.9% | 0.001 |
| Marital status: not married | 27.0% | 22.3% | 38.7% | <0.001 |
| Working status: not working | 77.8% | 71.6% | 74.4% | <0.001 |
| Self-perceived economic status: poor | 51.2% | 50.2% | 55.4% | 0.415 |
| Body mass index |  |  |  |  |
| Normal | 74.3% | 72.7% | 71.4% | 0.063 |
| Underweight | 6.7% | 6.6% | 11.9% |  |
| Overweight | 19.0% | 20.7% | 16.7% |  |
| Hypertension: present | 40.1% | 38.4% | 39.9% | 0.457 |
| Diabetes mellitus: present | 10.7% | 12.8% | 12.5% | 0.056 |
| Cerebrovascular disease: present | 2.7% | 2.6% | 2.4% | 0.989 |
| Cancer: present | 2.9% | 3.2% | 5.4% | 0.152 |
| Alcohol intake: daily/occasional drinkers | 35.2% | 43.6% | 29.8% | <0.001 |
| Smoking history: ex/current smokers | 32.1% | 44.3% | 35.7% | <0.001 |
| Eating habits: low dietary variety | 41.0% | 44.7% | 46.4% | 0.015 |
| Subjects with poor basic activities of daily living | 19.5% | 18.2% | 19.0% | 0.509 |
| Subjects with poor cognitive functioning | 13.8% | 13.0% | 17.9% | 0.207 |
| Subjects with depression | 19.9% | 18.5% | 27.4% | 0.020 |
| Subjects with poor self-rated health | 12.7% | 12.0% | 19.0% | 0.042 |
| Participation in social activities: none | 28.5% | 32.3% | 45.8% | <0.001 |

^a^ Fisher's exact test.
